# The Fertility Market in Latin America and Brazil: A Narrative Review of Trends and Key Players

**DOI:** 10.5935/1518-0557.20250025

**Published:** 2025

**Authors:** Maryana T. F. Câmara de Oliveira, Beatriz H. D. Rodrigues de Albuquerque, Janaína F. Aderaldo, Katrine B. Cavalcanti, Daniel C.F. Lanza

**Affiliations:** 1 Applied Molecular Biology Laboratory (LAPLIC). Federal University of Rio Grande do Norte, Brazil; 2 Graduate Program in Biochemistry and Molecular Biology (PPGBqBM). Federal University of Rio Grande do Norte, Brazil; 3 Januário Cicco Maternity School. Federal University of Rio Grande do Norte, Brazil

**Keywords:** fertility market, assisted reproduction, infertility, IVF, human reproduction

## Abstract

Facing a global increase in infertility rates, this study casts a spotlight on the fertility and assisted reproduction (ART) market within Brazil and Latin America, against a backdrop of considerable political and programmatic challenges. Addressing the scarcity of comprehensive studies and the variable accessibility to ART services, our objective is to unravel the growth dynamics and the disparities in ART accessibility and quality across these regions. Studies were analyzed within the PubMed, Google Scholar, and Grey Literature databases. Records retrieved from all the searches underwent screening based on title, abstract, and full-text examination. Inclusion criteria involved assessing titles, abstracts, and full texts of articles published within the past 10 years. Our study demonstrated growth in the opening of clinics and assisted reproduction centers and the performance of ART procedures in Brazil and Latin America. Despite overall low utilization rates, Brazil emerges as a pivotal contributor to the ART sector in the region. However, significant barriers such as high costs and limited insurance coverage impede access to ART treatments, underscoring the urgent need for strategic investments in research, development, and policy reform. The study underscores the critical demand for targeted interventions to enhance both accessibility and financial affordability of ART. By highlighting Brazil’s role and prospective market opportunities, it calls for a collaborative approach among stakeholders to foster an enabling environment for infertility treatment, promising to stimulate new advancements and market expansion in the coming years.

## INTRODUCTION

The World Health Organization (WHO) defines infertility as a condition of the reproductive system that affects both males and females, characterized by the inability to achieve pregnancy after 12 months or more of regular, unprotected sexual intercourse (WHO, 2023). Globally, approximately 1 in 6 adults experience infertility during their reproductive life, representing about 17.5% of the adult population, the prevalence is relatively consistent across continents, ranging from 16% to 18% in regions such as Africa, Asia, Europe, and the Americas (WHO, 2023). However, in developing countries, infertility rates are slightly higher, influenced by biological and socioeconomic factors that impact reproductive health (Vander Borght & Wyns, 2018; Aitken, 2024).

To mitigate this growing public health issue, Assisted Reproductive Technologies (ART) have become a crucial solution, encompassing a range of techniques designed to aid individuals and couples in achieving pregnancy (Patrizio *et al.*, 2022a, 2022b). As ART technology evolves, its application has expanded beyond infertile couples to include fertility preservation and fertile individuals planning their future (Doody, 2021). Among these techniques, in vitro fertilization (IVF) remains the most widely utilized, accounting for approximately 91.9% of the global fertility market revenue in 2022 (Markets and Markets, 2022).

In Latin America, the Latin American Network of Assisted Reproduction (RedLara) has been a key organization since 1990, collecting and analyzing data on ART outcomes through its Latin American Registry of Assisted Reproduction (RLA) (downloadable from www.redlara.com). The registry provides comprehensive reports on treatment metrics, clinic distribution, pregnancy rates, and live births across the region (Zegers-Hochschild *et al.*, 2021). In Brazil, the National Embryo Production System (SisEmbrio), regulated by RDC No. 29 of May 12, 2008, serves a similar function, tracking ART data to monitor the sector’s growth and ensure compliance with national guidelines (Anvisa - Agência Nacional de Vigilância Sanitária, 2008; Zegers-Hochschild *et al.*, 2025).

The global fertility services market is expanding rapidly, reaching $17.45 billion in 2021. It is projected to grow to $31.59 billion by 2029, with a compound annual growth rate (CAGR) of 7.70% (Data Bridge Market Research, 2022). Despite this growth, access to ART remains limited in LMICs, including Brazil, where the fertility market is still in its early stages of consolidation (Bourlon *et al.*, 2020; Rashedi *et al.*, 2020).

In terms of market distribution, Latin America, the Middle East, and Africa collectively account for only 10% of the global ART market, whereas Europe and North America dominate with around 65%, followed by the Asia-Pacific region at approximately 25% (Aderaldo *et al.*, 2023). Despite its smaller share, Brazil stands out within Latin America, recording 83,000 births from ART treatments in recent years, followed by Argentina with 39,366 births and Mexico with 31,903 births (RedLara, 2021; SBRA - Associação Brasileira de Reprodução Assistida, 2023; Zegers-Hochschild *et al.*, 2024).

Although Brazil leads Latin America in ART, political, economic, and ethical challenges significantly hinder the expansion of fertility services, particularly in less developed regions (Chiware *et al.*, 2021; Oliveira *et al.*, 2021). The concentration of ART clinics in more affluent areas exacerbates disparities in access, leaving many regions underserved (Oliveira *et al.*, 2021).

In this context, understanding the fertility market in Latin America and Brazil is essential for guiding technological innovation, expanding access to ART, and fostering investment in fertility services. This study aims to fill this gap by examining the key characteristics of the fertility market in these regions and highlighting the primary organizations driving its development.

## METHODS

This review is intended to discuss the relevant literature that has studied the development and access to fertility care services in Brazil and Latin America. Our search was conducted between September 2022 and August 2024 in the databases PubMed (US National Library of Medicine), Google Scholar, and gray literature. This was followed by the selection of search terms to optimize information gathering and identify relevant records. The following search MeSH terms were used on PubMed: (assisted reproductive market) AND (fertility market) OR (infertility market). The search was not limited by language and included articles published in the last 10 years. On Google Scholar, the search strategy involved the MeSH terms: “fertility” AND “assisted reproduction” AND “market” AND “Latin America”, with the results limited to publications between 2014 and 2024. Initially were considered journal articles, market reports, academic productions, and documents from indexed government agencies. To supplement the data, an additional analysis of records including other sources was manually conducted. The records retrieved from all searches underwent screening by title, abstract, and full text. Inclusion criteria analyzed titles, abstracts, and full texts of articles in the last 10 years addressing the assisted reproduction market, fertility market, and use of assisted reproductive technologies in Latin America and Brazil. Exclusion criteria were duplicates (citations present in more than one database), book chapters, and titles, abstracts, and full texts not discussing the topics ranked in the inclusion criteria. Selected articles have been read by at least two researchers. The review was aimed to find answers to the following questions: What are the trends in the fertility and assisted reproduction market in Latin America and Brazil? And who are the main participants in offering services?

## RESULTS

### General Data Characteristics

**Figure 1:**
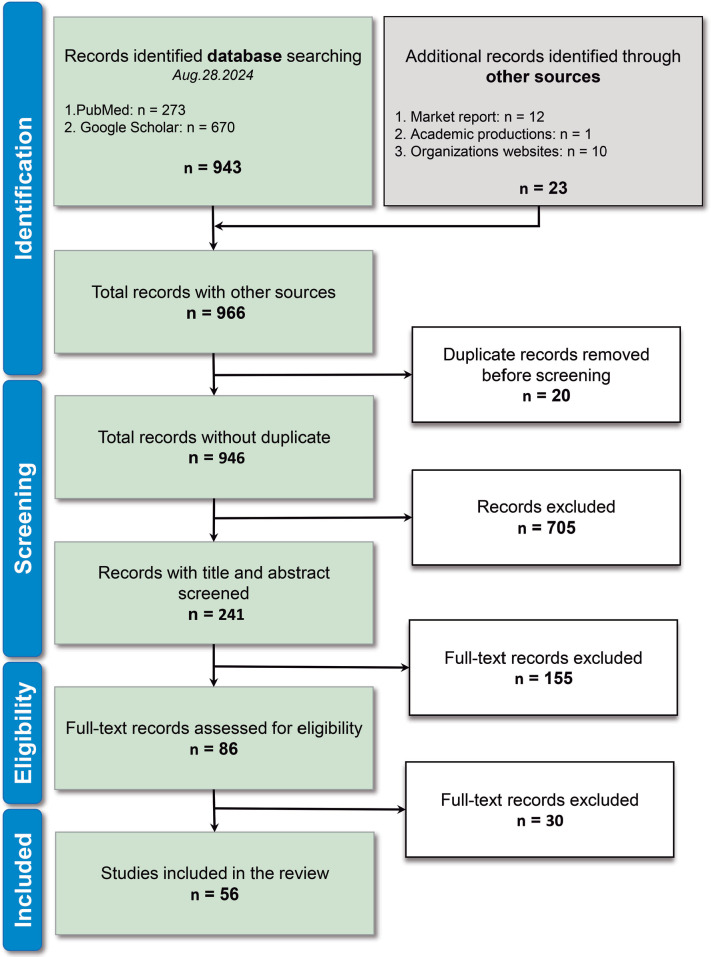
Flowchart of the identification and selection of studies through database searches.

The literature search concluded on August 28, 2024, yielding a total of 966 records. From database searches, 273 records were retrieved from PubMed and 670 from Google Scholar, totaling 943 records, with the remaining 23 records obtained from gray literature. Initially, 20 records duplicate removal were excluded, and 946 unique records were screened. Title and abstract screening led to the exclusion of 705 records not directly related to the topic, resulting in 241 records for full-text review. After full-text analysis, 155 records were excluded for not finding inclusion criteria, leaving 86 records assessed for eligibility. In the final stage, 56 records were included in this review, comprising 33 scientific articles, 12 market reports, 1 academic production, and 10 government organization documents (Fig. 1).

### Assisted Reproduction Market in Latin America

In Latin America, infertility significantly impacts with estimates suggesting a prevalence of 10% to 20% among couples of reproductive ages (INEGI, 2020). This region is experiencing steady growth in the fertility market projecting revenues of $6.6 billion by 2021 with a CAGR of over 8% (Salama *et al.*, 2018; Zegers-Hochschild *et al.*, 2019a, 2019b).

According to the RLA of RedLara, 204 centers across 16 countries submitted data on ART procedures in 2021 (Zegers-Hochschild *et al.*, 2025). It is important to note that this number reflects only the centers that provided data to the registry and not the total number of assisted reproduction centers in Latin America. The most recent report documented 127,351 initiated cycles in 2021, representing a 31% increase compared to 2020 (Zegers-Hochschild *et al.*, 2025).

**Figure 2:**
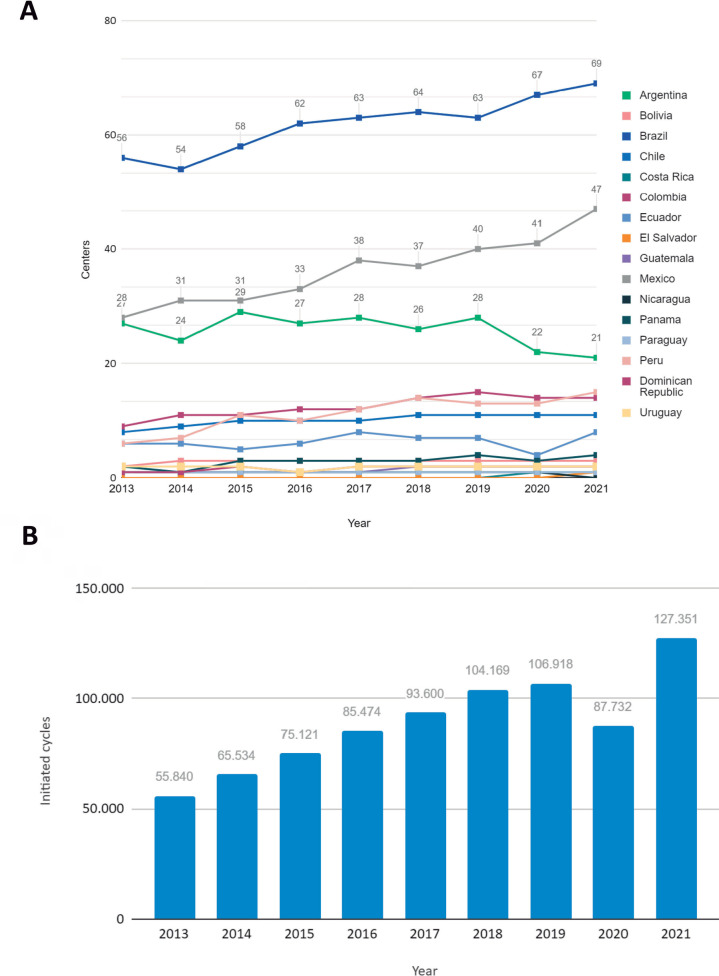
ART in Latin America over the years. A) Distribution of assisted reproduction centers among the countries of Latin America between the years 2013 to 2021. B) Cycles conducted with ART in Latin America between the years 2013 to 2021.

Over recent years Brazil has led the number of assisted reproduction centers in Latin America totaling 69 centers cited in the last report followed by Mexico with 47 and Argentina with 21 centers (Zegers-Hochschild *et al.*, 2016, 2017, 2019a, 2019b, 2020, 2021, 2022, 2024, 2025) (Fig. 2A). Between 2013 and 2021 Latin America conducted 807.739 ART cycles with growth until 2019 (Zegers-Hochschild *et al.*, 2022). In the pandemic year, there were 87,732 cycles, a decrease of 19.186 from the previous year (Zegers-Hochschild *et al.*, 2022, 2024) (Fig. 2B).

**Figure 3:**
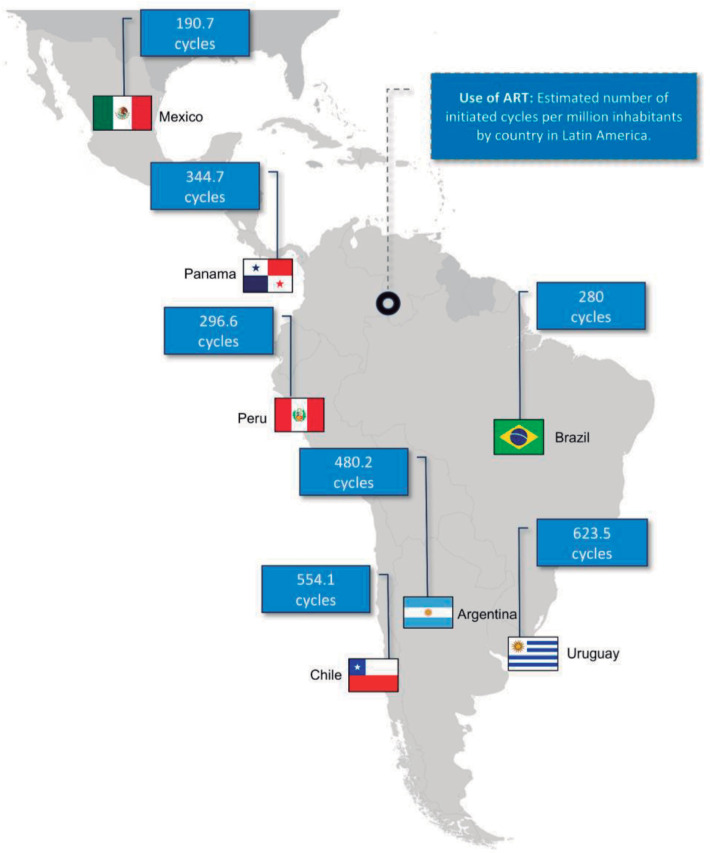
Use of ART in the main countries of Latin America in the year 2021. The figure includes data only for countries where the ART utilization rate exceeds 150 cycles/million. The figure includes data only for countries where the ART utilization rate exceeds 150 cycles/million.

Figure 3 illustrates the Latin American countries with the highest proportional rates of ART use per inhabitant. The data represented in the figure includes only countries exceeding a cut-off value of 150 cycles per million inhabitants, this cut-off value was applied to focus on regions with relatively higher adoption of ART. Regarding the estimated number of cycles initiated per million inhabitants in Latin America, Uruguay and Chile lead recording 623.5 and 554.1 cycles performed per million inhabitants respectively (Zegers-Hochschild *et al.*, 2022). Despite Brazil leading in the number of cycles performed, the rate of ART use is still low with 280 cycles per million inhabitants.

**Figure 4:**
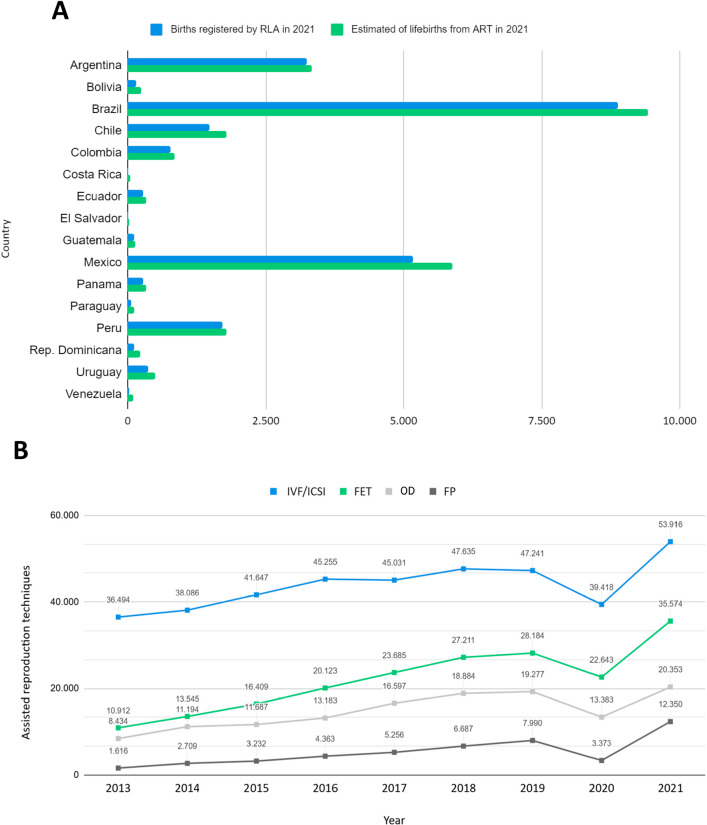
Performance of ART in Latin America. A) Estimated and registered births in the year 2021. B) ART treatments reported in the years 2013 to 2021. FET = autologous frozen embryo transfer; FP = fertility preservation; IVF = *In vitro* fertilization; ICSI = intracytoplasmic sperm injection; OD = oocyte donation with fresh or frozen/thawed embryos.

Despite low ART use, Brazil led in births in 2021 (n=8.872) followed by Mexico (n=5.169) and Argentina (n=3.246) (Zegers-Hochschild *et al.*, 2025) remaining leaders in assisted human reproduction procedures (Fig. 4A).

Of the assisted reproduction techniques used in treatments from 2013 to 2021, 394.723 (51.03%) were in vitro fertilization/intracytoplasmic sperm injection (IVF/ICSI), 198.286 (25.63%) autologous frozen embryo transfer (FET), 132.992 (17.19%) oocyte donation (OD), and 47.576 (6.15%) fertility preservation (FP) (Zegers-Hochschild *et al.*, 2016, 2017, 2019a, 2019b, 2020, 2021, 2022, 2025). Figure 4B shows the growth in the use of these procedures over the last few years.

### Assisted Reproduction Market in Brazil

Approximately 8 million individuals in Brazil are infertile and the national assisted reproduction market is already valued at R$1.3 billion, underscoring its significance (IVF Brazil, 2022; SBRA - Associação Brasileira de Reprodução Assistida, 2023). It is estimated that between 2021 and 2025 around 77.588 patients up to 35 years old will be treated via IVF in Brazil (Lakryc *et al.*, 2019). Compared to developed countries which experience ART growth between 5% and 10% Brazil boasts a 16% CAGR and the fertility market is expected to triple until 2030 (IVF Brazil, 2022). Data from SisEmbrio indicates that the assisted reproduction market grew from 24.147 treatments in 2013 to 56.624 in 2023 (Anvisa - Agência Nacional de Vigilância Sanitária, 2024).

According to RedLara, Brazil is home to approximately 40% of assisted reproduction clinics in Latin America in terms of infrastructure, such as physical and operational capacity, covering facilities, equipment, and resources needed to provide assisted reproduction services (Zegers-Hochschild *et al.*, 2019a, 2019b). SisEmbrio data reveal significant growth in clinics across Brazilian states. Brazil has 189 active human-assisted reproduction centers, with 120 more centers concerning the REDLARA data, doubling the number of centers since 2013 (Supplementary Material 1) (Anvisa - Agência Nacional de Vigilância Sanitária, 2024).

Brazil is geographically divided into five regions: North, Northeast, Central-West, Southeast, and South, comprising a total of 26 states and one Federal District. Regarding the distribution of ART centers across the country, the Southeast region hosts the majority, with 55.55% of the clinics. Within this region, São Paulo (SP) leads with 63 clinics, followed by Minas Gerais (MG) with 24 clinics (Anvisa - Agência Nacional de Vigilância Sanitária, 2014, 2015, 2016, 2017, 2018, 2019, 2020, 2022, 2024).

**Figure 5:**
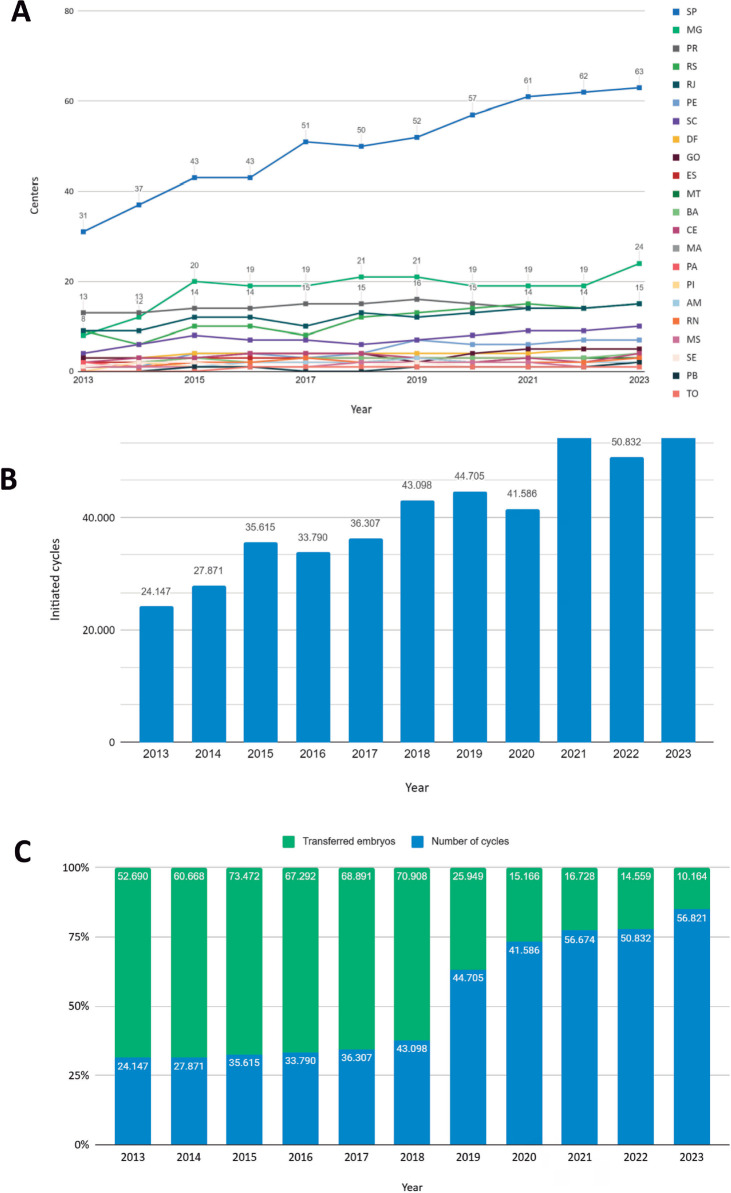
ART in Brazil over the years. A) Distribution of assisted human reproduction centers in Brazil over the last 10 years. B) Number of cycles with ART conducted in Brazil over the last 10 years. C) Number of cycles conducted with ART and embryos transferred between the years 2013 to 2023.

The South region ranks second, accounting for 21.16% of ART centers, followed by the Northeast region with 13.22%. The Central-West and North regions have the fewest clinics, contributing 7.40% and 2.60% of the total, respectively, highlighting disparities in access to fertility care across Brazil (Fig. 5A).

Between 2013 and 2023, 2023 recorded the highest number of cycles with a total of 56,821 cycles initiated, an increase of 5.989 cycles compared to the previous year (Fig 5B). In 2020 there was a decrease in cycles in the pandemic scenario, but a significant increase in the number of cycles in the following years was observed with a return to normal operation after the pandemic (Anvisa - Agência Nacional de Vigilância Sanitária, 2024).

In 2023, a total of 10.164 embryos were transferred while 56.821 cycles were performed (Anvisa - Agência Nacional de Vigilância Sanitária, 2024). It was observed that the number of transferred embryos fell by almost 80% in recent years, while the number of performed cycles showed steady development (Fig. 5C).

The number of frozen embryos in Brazil doubled between 2013 and 2021 yet there was a drop in 2020 and 2022 as shown in Table 1. Regarding frozen embryos in 2022, the largest proportion (70%) out of a total of 97.334 embryos is concentrated in the Southeast region of the country (Anvisa - Agência Nacional de Vigilância Sanitária, 2014, 2015, 2016, 2017, 2018, 2019, 2020, 2022). Among the states, São Paulo leads with the highest number of frozen embryos (SP=53,076) followed by Minas Gerais (MG=5,830) and Rio de Janeiro (RJ=5,160) (Anvisa - Agência Nacional de Vigilância Sanitária, 2022) (Table 1).

**Table 1:** Data on the average fertilization rate, frozen embryos, and discarded embryos among the states of Brazil in 2023.

Regions	States (UF)	Frozen embryos	Discarded embryos	Average fertilization rate
**North**	Amazonas (AM)	237	148	69%
	Pará (PA)	714	346	71%
	Tocantins (TO)	160	37	85%^*^
**Northeast**	Bahia (BA)	2,524	1,248	79%
	Ceará (CE)	2,765	1,583	74%
	Maranhão (MA)	652	341	73%^*^
	Paraíba (PB)	837	178	78%
	Pernambuco (PE)	3,124	1,597	80%
	Piauí (PI)	1,315	593	75%
	Rio Grande do Norte (RN)	618	326	49%^*^
	Sergipe (SE)	699	566	78%
**Central-West**	Distrito Federal (DF)	2,618	1,255	79%
	Goiás (GO)	4,628	2,175	79%
	Mato Grosso (MT)	959	597	79%
	Mato Grosso do Sul (MS)	324	109	75%^*^
**Southeast**	Espírito Santo (ES)	1,281	610	61%
	Minas Gerais (MG)	8,634	4,551	71%
	Rio de Janeiro (RJ)	7,641	4,036	79%
	São Paulo (SP)	61,742	27,123	76%
**South**	Paraná (PR)	4,211	2,033	73%
	Rio Grande do Sul (RS)	7,345	3,744	78%
	Santa Catarina (SC)	2,717	1,235	81%
**Total**		**115,745**	**54,431**	**75%^†^**

* Incomplete data.

† National average.

Regarding frozen and discarded embryos in 2023, the most populous states in the country lead with the highest number, as shown in Table 1. The South region had the highest rate, with 78%, followed by the Central-West region, with 75%. On the other hand, the Northeast region had the lowest fertilization rate, with 72.75% (Table 1).

## DISCUSSION

Globally the use of the fertility and assisted reproduction market is driven by the rising prevalence of infertility varying with public policies and access to infertility treatments in each country (Adamson *et al.*, 2023). Despite advances in the fertility and assisted reproduction market, access to reproductive health services and infertility treatments still presents challenges in several Latin American countries, including Brazil (Torres *et al.*, 2019; Machin *et al.*, 2020).

Reports published by REDLARA since 2013 demonstrate growth in the number of assisted reproduction centers and, consequently, in the number of cycles performed (Fig. 2A). RedLara plays a key role in advancing the field of ART in Latin America with a rigorous accreditation process (rigorous evaluation of clinical protocols, laboratory practices, patient care procedures, and ethical guidelines), contributing significantly to the development and standardization of ART in Latin America (RedLara - Red Latinoamericana de Reproducción Asistida, 2024).

Following the pandemic, most countries saw a significant rise in fertility centers, initiated cycles, and births. The post-COVID effect observed through preliminary 2021 national data reported by U.S. clinics to the Society for Assisted Reproductive Technology (SART, 2023) further strengthens the case for the decline in procedures in 2020, like data from Latin American countries (Fig. 2B).

In the context of the fertility market in Latin America, due to social issues and especially the higher costs of ART procedures, many individuals tend to seek medical treatments abroad, through cross-border reproductive care (CBRC) or fertility tourism (Salama *et al.*, 2018; de Mouzon *et al.*, 2020). This behavior further centralizes the trade in Europe and North America, which account for approximately 67% of the global ART market (Data Bridge Market Research, 2022).

The scarcity of specialized clinics in some countries, high costs, and lack of insurance coverage are significant barriers to accessing these services (Inhorn *et al.*, 2018). In addition, moral and religious considerations also affect the uptake of fertility treatments in the region, resulting in a relatively low adoption rate compared to other parts of the world (Patrizio *et al.*, 2022a, 2022b).

Regarding ART utilization in Latin America in 2021, Uruguay leads in ART utilization, with 623.5 cycles per million, driven by a universal care law and a strong state financing program. With a government program covering half of the fixed cost, Chile increased ART utilization from 372 to 554.1 cycles per million between 2019 and 2021. Despite universal care legislation in Argentina, utilization fell from 490 to 480.2 cycles per million over the same period due to economic hardship. These fluctuations reflect the impact of access to financing, especially in countries where the burden falls on patients (Fig. 3).

Global ART utilization rate is 535 cycles per million inhabitants significantly varying among countries (16 to 5,203 cycles) (Chambers *et al.*, 2021). The average number of ART cycles per million in 15 Latin American countries (204 cycles/million) is just 14.6% of the average utilization of 1,400 cycles per million in 21 European countries (European IVF-monitoring Consortium (EIM)‡ for the European Society of Human Reproduction and Embryology (ESHRE) *et al.*, 2020). However, despite the economic lag of the region compared to the rest of the world, our data indicate that the use of ART in Latin America is growing (Inhorn *et al.*, 2018).

Chile, Uruguay, and Panama showed low registered births because they have smaller territories, despite being among the countries that use ART the most. On the other hand, despite having the highest number of births, Brazil needs to consider that the use of ART is still low and has a lot of territory to explore and expand access to these technologies (Fig. 4A).

Regarding the ART used in recent years, fresh IVF and ICSI cycles accounted for 42.3% of initiated cycles, followed by 27.9% of FET, an increase from the 25% observed in 2020 (Fig. 4B). The share of cycles with oocyte donation remained high (16%), significantly above the approximately 7.6% recorded in European countries (European IVF Monitoring Consortium (EIM) for the European Society of Human Reproduction and Embryology (ESHRE), 2023).

In the context of the Brazilian fertility market, the use of ART in the country currently stands at just 231 cycles per million inhabitants. According to the European Society of Human Reproduction and Embryology (ESHRE), the ideal would be 1.000 treatment cycles per million inhabitants (Guideline Group on Unexplained Infertility, 2023), which reflects that this market has significant potential for development (Zegers-Hochschild *et al.*, 2024). Despite low ART utilization, Brazil significantly contributes to the region’s number of births from ART and cycle performance (Fig. 4A).

Regarding the distribution of reproductive centers and the provision of ART services, we observed that the vast majority are in the South and Southeast regions of Brazil (Fig. 5A and Supplementary Material 1). We attribute this discrepancy in proportion to the economy of the region where the largest offer of technologies and opportunities is centralized in the country’s more developed regions, inducing the migration of individuals seeking uncomplicated access to treatments not available in their residential areas, the panorama is like what is observed in other studies, including on a global scale (Silva *et al.*, 2019; Lass & Lass, 2023).

For initiated cycles, Brazil has shown variations over the years. In 2020 the country also experienced a decrease in IVF cycles and ART procedures due to COVID-19, dropping from 44,705 cycles in 2019 to 33,058. Despite this, with the post-pandemic resumption, data indicate that the number of cycles began to increase again in 2021, as shown in Fig. 5B.

The level of Latin America, Brazil led the region, accounting for 44.2% of all cycles, followed by Argentina and Mexico, with 16.8% and 16.7%, respectively (Zegers-Hochschild *et al.*, 2025). Brazil’s prominence in Latin America’s fertility market is driven by a trend of delayed first pregnancies, influenced by modern reproductive behaviors such as longer life expectancy, contraception use, socioeconomic factors, and societal changes (Goisis *et al.*, 2020; Oliveira *et al.*, 2021).

The main barrier to accessing ART treatments is the lack of insurance coverage combined with the high cost of treatments, which are inaccessible to most people (Salama *et al.*, 2018). Only a small number of assisted reproduction treatment centers are covered by Brazil’s Unified Health System (SUS) and in most of them, users must pay at least part of the costs (Entringer *et al.*, 2023). The cost per procedure for high-complexity assisted reproduction in Brazil varies by technique. For IVF with ICSI, the cost is approximately USD 3.730, for conventional IVF, it is about USD 2.985, and for artificial insemination, the cost is around USD 1.120 (Entringer *et al.*, 2023). These values are based on exchange rates of BRL to USD at the time of reporting and may vary depending on currency fluctuations. Thus, the cost of undergoing ART treatments remains discordant with the financial situation of most of the Brazilian population (Machin *et al.*, 2020).

The observed data also present a significant change in the number of embryos transferred compared to the number of cycles performed in recent years in Brazil (Fig. 5C). This strongly correlates with the recent research and introduction of more effective methods for selecting healthy embryos before transfer to the uterus, as well as the expansion of Preimplantation Genetic Testing (PGT) allowing genetic analysis of embryos before transferring to the patient (Madero *et al.*, 2023).

In the state of São Paulo, 61,742 embryos were frozen in 2021. These numbers were much higher when compared to other more populous states (Anvisa - Agência Nacional de Vigilância Sanitária, 2024). Minas Gerais froze 8,634 embryos, and Rio de Janeiro had a similar number, with 7,641 frozen embryos. In addition, São Paulo discarded 27,123 embryos, far exceeding the numbers of Minas Gerais (n=4,551) and Rio de Janeiro (n=4,036). It is important to consider the centralization and demand for these services in the state of São Paulo, however, ART services need to expand and access other regions of the country.

Regarding the average fertilization rate (ratio of fertilized oocytes to the number of inseminated oocytes), data from SisEmbrio from 2024 indicate that the national average was 75% (Anvisa - Agência Nacional de Vigilância Sanitária, 2024). Generally, the states have similar fertilization rates, but some states have incomplete data in SisEmbrio.

Our research identified the main Latin American countries and Brazilian states offering fertility services and available techniques. However, to attract more patients and accurately report outcomes, clinics must establish consistent data registration across Latin America, enabling public policies to support service growth and access.

In the roadmap for the expansion of the fertility market in the country, it is also important to promote the decentralization of human reproduction, with the opening of clinics in regions outside major population centers and in the five Brazilian states that still do not have clinics with embryology laboratories, such as Alagoas, Rondônia, Roraima, Acre, and Amapá (Anvisa - Agência Nacional de Vigilância Sanitária, 2023).

### Research Gaps and Challenges of the Analysis

The analysis highlights significant gaps in studies on fertility and assisted reproduction markets, especially in certain regions, complicating comparisons across Latin America. Most available studies provided only partial insights, often requiring paid access to more detailed information. The estimates primarily focused on market value and segmentation, derived from market reports and government data.

Comprehensive research is needed to produce robust estimates of market values and investment opportunities, as publicly accessible data often lacks commercial metrics. Consistent and systematic data collection methods are essential to accurately assess and compare markets globally, regionally, and nationally. Additionally, policymakers should address cost barriers to ensure broader access to market information, and expanding research in academic settings is crucial to overcoming these limitations.

## CONCLUSIONS

The rising global incidence of infertility, combined with advancements in the fertility market, is expected to drive further growth in the coming years. Despite ongoing political and programmatic challenges related to limited availability, accessibility, and quality of infertility treatments in many countries, our study showed a rise in the number of clinics and Assisted Reproduction centers, as well as an increase in ART procedures in Brazil and Latin America, with potential for further expansion and decentralization. This projected growth emphasizes the need for continued investment in research and development, while also presenting significant business opportunities for companies in the fertility and assisted reproduction sector.

## Appendix

**Supplementary Material 1:** Centers distributed among the states of Brazil. São Paulo: SP / Minas Gerais: MG / Rio de Janeiro: RJ / Espírito Santo: ES / Rio Grande do Sul: RS / Paraná: PR / Santa Catarina: SC / Distrito Federal: DF / Goiás: GO / Mato Grosso do Sul: MS / Mato Grosso: MT / Bahia: BA / Ceará: CE / Pernambuco: PE / Paraíba: PB / Piauí: PI / Maranhão: MA / Rio Grande do Norte: RN / Sergipe: SE / Amazonas: MA / Pará: PA / Tocantins: TO.

States (UF)	Centers	Latin American Assisted Reproduction Registry (RLA)	Data in the SisEmbrio (ANVISA)
SP	ANDROFERT - Clínica de Andrologia e Reprodução Humana	Yes	Yes
SP	Art Medicina - Reprodução Humana Assistida	No	Yes
SP	CEERH - Centro Especializado em Reprodução Humana	Yes	No
SP	CEFERP Reprodução Assistida	No	Yes
SP	Célula Mater Saúde da Mulher	No	Yes
SP	Chedid Grieco Medicina Reprodutiva	No	Yes
SP	Centro de Reprodução do Estado da Saúde de São Paulo	No	Yes
SP	Centro de Reprodução Humana de Piracicaba	No	Yes
SP	Centro de Reprodução Humana de São José do Rio Preto	Yes	Yes
SP	Centro de Reprodução Humana FIV Marília	No	Yes
SP	Centro de Reprodução Humana Monteleone	Yes	No
SP	Centro de Reprodução Humana Prof. Franco Junior	Yes	Yes
SP	Centro de Reprodução Humana Wahib Hassan	Yes	Yes
SP	Centro de Reprodução Humana do Hospital e Maternidade Santa Joana	Yes	Yes
SP	CITI - Centro de Investigação e Tratamento da Infertilidade	No	Yes
SP	Clínica Ana Bartmann	No	Yes
SP	Clínica Dr. José Bento	No	Yes
SP	Clínica Endogin Serh	No	Yes
SP	Clínica Fertilidade&Vida	No	Yes
SP	Clínica Fértilis de Medicina Reprodutiva	No	Yes
SP	Clínica Invitro RA	Yes	No
SP	Clínica La Vie - Franca	No	Yes
SP	Clínica Matrix	No	Yes
SP	Clínica Progerar Saúde e Reprodução Humana	Yes	No
SP	Clínica Vida Medicina Reprodutiva	Yes	No
SP	Embriocare - Reprodução Assistida e Medicina Fetal	No	Yes
SP	Embriofert Clínica Médica Hospitalar - Fertility Medical Group	Yes	Yes
SP	Embryo Fetus - Centro de Reprodução Humana e Medicina Fetal	No	Yes
SP	Embryolife - Instituto de Medicina Reprodutiva	Yes	Yes
SP	Engravida Clínica de Reprodução Humana - São Paulo	No	Yes
SP	Fert-Embryo - Centro de Medicina Reprodutiva	No	Yes
SP	FERTICLIN - Clínica de Fertilidade Humana	Yes	Yes
SP	FERTILITY - Centro de Fertilização Assistida	Yes	Yes
SP	FERTIVITRO - Centro de Reprodução Humana	Yes	Yes
SP	FIVMED - Instituto de Reprodução Humana	Yes	No
SP	Fleury Centro de Medicina Reprodutiva	No	Yes
SP	Genics Medicina Reprodutiva e Genômica	Yes	Yes
SP	GERA - Grupo de Endoscopia e Reprodução Assistida	Yes	Yes
SP	Hospital das Clínicas Ribeirao Preto - Reprodução Humana	Yes	Yes
SP	Hospital Sírio-Libanês	No	Yes
SP	Huntington Centro de Medicina Reprodutiva - Campinas	Yes	Yes
SP	Huntington Centro de Medicina Reprodutiva - Ibirapuera	Yes	Yes
SP	Huntington Centro de Medicina Reprodutiva - Vila Mariana	Yes	Yes
SP	InVentre Centro Avançado de Medicina Reprodutiva	No	Yes
SP	Instituto Ideia Fértil de Saúde Reprodutiva - Unidade Santo André	Yes	Yes
SP	Instituto Ideia Fértil de Saúde Reprodutiva - Unidade São Paulo	Yes	Yes
SP	Lab For Life Centro de Medicina Reprodutiva	Yes	Yes
SP	Mater Lab Medicina Reprodutiva	No	Yes
SP	Nilo Frantz Medicina Reprodutiva	No	Yes
SP	Núcleo Santista de Reprodução Humana	No	Yes
SP	Originare - Centro de Reprodução Humana	Yes	Yes
SP	Reproduce Laboratório de Reprodução Assistida	No	Yes
SP	Reproduction Reprodução Humana Assistida	No	Yes
SP	Reproduh Centro de Reprodução Humana	No	Yes
SP	Reproferty Centro de Reprodução Humana - Saúde da Mulher e Saúde do Homem	Yes	No
SP	Semear Fertilidade - Reprodução Humana	No	Yes
SP	Sesma Clínica de Reprodução Humana	No	Yes
SP	SPDM - Associação Paulista para o Desenvolvimento da Medicina	No	Yes
MG	Cegonha Medicina Reprodutiva	Yes	Yes
MG	Clínica La Vie - Uberaba	No	Yes
MG	Clínica Ovular Fertilidade e Menopausa	No	Yes
MG	Clínica Vilara - Centro de Medicina Reprodutiva no Sul de Minas	No	Yes
MG	Clínica Vilara - Hospital Vila Da Serra	No	Yes
MG	Clínica Vita - Reprodução Humana e Cirurgia Ginecológica	No	Yes
MG	Engravida Clínica de Reprodução Humana - Juíz de Fora	No	Yes
MG	Fecunda Reprodução Humana	Yes	Yes
MG	Fertibaby Medicina Reprodutiva	Yes	Yes
MG	Fértil Reprodução Humana	Yes	Yes
MG	Fertminas Muriaé - Medicina Reprodutiva	No	Yes
MG	Gerar *In Vitro* - Reprodução Assistida	No	Yes
MG	Hospital das Clínicas da Universidade Federal de Minas Gerais - HC-UFMG	No	Yes
MG	Huntington Pró-Criar Medicina Reprodutiva	Yes	Yes
MG	IBRRA - Instituto Brasileiro de Reprodução Assistida	No	Yes
MG	LIFE Reprodução Humana	No	Yes
MG	NIDUS Medicina Reprodutiva	Yes	Yes
MG	Origen - Centro de Medicina Reprodutiva	Yes	No
MG	Reprodução Humana Mater Dei	No	Yes
RJ	Clínica Dale - Day Clinic Rio Sul	Yes	No
RJ	Clínica Fertil - Medicina Reprodutiva	No	Yes
RJ	Clínica Gerar Vida Medicina Reprodutiva	Yes	Yes
RJ	Clínica Vida Medicina Reprodutiva	Yes	Yes
RJ	Embrion - Medicina Reprodutiva	No	Yes
RJ	Engravida Clínica de Reprodução Humana - Rio de Janeiro	No	Yes
RJ	Fertilizare - Medicina Reprodutiva	No	Yes
RJ	FERTIPRAXIS - Centro de Reprodução Humana	Yes	Yes
RJ	Grupo Pró-Fértil Medicina Reprodutiva	No	Yes
RJ	Humana Medicina Reprodutiva	No	Yes
RJ	OrigenRio - Medicina Reprodutiva	Yes	Yes
RJ	Primordia - Medicina Reprodutiva	Yes	No
RJ	Pró Nascer - Clínica de Reprodução Humana	Yes	Yes
ES	Biofert - Centro de Reprodução Humana	No	Yes
ES	Jules White - Medicina Reprodutiva	Yes	Yes
ES	Unifert - Centro Avançado de Reprodução Humana	No	Yes
RS	Centro de Fertilidade do Hospital Moinhos de Vento	Yes	No
RS	Centro de Reprodução Humana Bruno Born	No	Yes
RS	CONCEPTION - Centro de Reprodução Humana	Yes	No
RS	Embrios - Centro de Reprodução Humana	Yes	Yes
RS	Fertilitat - Centro de Medicina Reprodutiva	Yes	Yes
RS	Generar Reprodução Humana	Yes	Yes
RS	Genesis - Centro de Reprodução Humana	Yes	Yes
RS	Gerarte - Clínica de Reprodução Humana	No	Yes
RS	Hospital de Clínicas de Porto Alegre	No	Yes
RS	Hospital Femina - Unidade de Reprodução Humana	No	Yes
RS	Insemine - Centro de Reprodução Humana	Yes	Yes
RS	Nilo Frantz Medicina Reprodutiva	Yes	No
RS	Nilo Frantz Medicina Reprodutiva - Porto Alegre	No	Yes
RS	Proser - Clínica de Fertilidade e Reprodução Assistida	Yes	Yes
PR	Androlab - Clínica da Fertilidade	Yes	Yes
PR	Centro de Fertilidade Saab	No	Yes
PR	CIM Centro Integrado da Mulher - Maringá	No	Yes
PR	Conceber - Centro De Reprodução Humana	Yes	Yes
PR	Creag - Centro de Reprodução Assistida de Gurapuava	No	Yes
PR	Embryo - Centro de Reprodução Humana	No	Yes
PR	Feliccità Instituto de Fertilidade	Yes	Yes
PR	Fertclínica - Centro de Fertilidade	No	Yes
PR	Fertway - Reprodução Humana	Yes	Yes
PR	Genesis - Centro de Assistência em Reprodução Humana	Yes	Yes
PR	Materbaby - Hospital Almodin	No	Yes
PR	Progênese - Clínica de Fertilidade e Vida Reprodutiva	No	Yes
SC	Clínica Crifert - Reprodução Humana	Yes	Yes
SC	Clínica Fertilizar - Centro Catarinense de Reprodução Humana	No	Yes
SC	Clínica Procriar MRD	Yes	Yes
SC	Clinifert - Centro de Reprodução Humana.	Yes	Yes
SC	Fecondare - Centro de Medicina Reprodutiva	No	Yes
SC	FertiBC - Centro de Reproduçao Assistida	No	Yes
SC	Fertvita Medicina Reprodutiva	No	Yes
SC	Gaia - Centro de Reprodução Assistida	No	Yes
SC	Geravita - Clínica de Reprodução Humana	No	Yes
SC	SATIS - Centro de Reprodução Assistida de Joinville	No	Yes
DF	Centro de Ensino e Pesquisa em Reprodução Assistida do Hospital Materno Infantil de Brasília - CEPRA HMIB	Yes	Yes
DF	Engravida Clínica de Reprodução Humana - Brasília	No	Yes
DF	Genesis - Centro de Assistência em Reprodução Humana	Yes	Yes
DF	Huntington Medicina Reprodutiva - Brasília	Yes	Yes
DF	Instituto Verhum Vídeo Endoscopia e Reprodução Humana	Yes	Yes
GO	Centro de Reprodução Assistida Femina Maternidade	No	Yes
GO	Fértile Reprodução Humana	Yes	Yes
GO	Hospital das Clínicas da Universidade Federal de Góias	No	Yes
GO	Humana Medicina Reprodutiva	Yes	No
GO	In Vitro Clínica de Reprodução Humana Assistida de Goiânia	No	Yes
GO	Perfetto Reprodução Humana	No	Yes
MS	Clínica Gera - Campo Grande	No	Yes
MS	FertLiv Reprodução Assistida	Yes	No
MT	Clínica Fecundar Medicina Reprodutiva	No	Yes
MT	INTRO - Instituto Tropical de Medicina Reprodutiva	No	Yes
MT	LIFE Reprodução Humana	Yes	Yes
BA	CENAFERT - Centro de Medicina Reprodutiva	Yes	Yes
BA	Centro de Reprodução Humana Mater Dei - Salvador	No	Yes
BA	Clínica de Reprodução Humana Bela Zausner	No	Yes
BA	IVI - Clínica de Reprodução Assistida e Fertilidade em Salvador	No	Yes
CE	Bios - Centro de Medicina Reprodutiva do Ceará	Yes	Yes
CE	Clínica Fertiliza Sobral	No	Yes
CE	CONCEPTUS - Centro de Reprodução Assistida do Ceará	Yes	Yes
CE	Fertibaby Ceará	No	Yes
PE	Art Fértil - Clínica de Reprodução Assistida	No	Yes
PE	Centro de Reprodução Humana de Pernambuco	No	Yes
PE	Clínica Arminio Mota Collier	No	Yes
PE	Clínica de Reprodução Humana de Petrolina	No	Yes
PE	GEARE Medicina Reprodutiva - Recife	Yes	Yes
PE	NASCER Medicina Reprodutiva	Yes	Yes
PB	Biofertil Instituto Médico de Reprodução Humana	No	Yes
PB	Geare Medicina Reprodutiva - João Pessoa	No	Yes
PI	Criar Vidas - Clínica de Reprodução Assistida	No	Yes
PI	Fertvida Prime	No	Yes
MA	Eva Medicina Reprodutiva	No	Yes
MA	Fertvida São Luís	No	Yes
RN	Bios - Centro de Medicina Reprodutiva	No	Yes
RN	DNA Fértil	No	Yes
RN	Empresa Brasileira De Servicos Hospitalares - Ebserh	No	Yes
SE	Fertilità - Clínica de Reprodução Humana	No	Yes
MA	Engravida Clínica de Reprodução Humana - Manaus	No	Yes
MA	La Vitta - Centro De Reprodução Humana	No	Yes
PA	Clínica Pronatus Medicina Reprodutiva	No	Yes
TO	Gerare Reprodução Humana	No	Yes
